# Economic Evaluation of Prevention Interventions for Child Sexual Exploitation or Child Sexual Abuse: A Systematic Review

**DOI:** 10.1177/15248380241284782

**Published:** 2024-10-10

**Authors:** Sithara Wanni Arachchige Dona, Genevieve Bloxsom, Julie Green, Mary Rose Angeles, Cathy Humphreys, Lisa Gold

**Affiliations:** 1 Deakin Health Economics, School of Health and Social Development, Institute for Health Transformation, Faculty of Health, Deakin University, Geelong, VIC, Australia; 2 Department of Social Work, The University of Melbourne, Parkville, VIC, Australia; 3 Cairns Community Legal Centre, QLD, Australia

**Keywords:** economic evaluation, cost-effectiveness, intervention, child sexual abuse, child sexual exploitation

## Abstract

Child sexual abuse, including sexual exploitation, is a global issue, affecting 8% to 31% of girls and 3% to 17% of boys worldwide. This systematic review aims to synthesize economic evidence on the cost-effectiveness of community interventions to prevent child sexual abuse/exploitation to inform decision-making. A systematic search was conducted on eight databases for studies published until April 2023. Gray literature was searched using Google. The inclusion criteria were economic evaluation of interventions targeted at children, perpetrators/offenders, or professionals addressing child sexual abuse/exploitation. There was no limitation by country, but an English language abstract was required for non-English articles. Studies without a specific focus on child sexual abuse/exploitation, such as physical, emotional, and domestic violence-related abuse, were excluded. All costs were adjusted to US$ 2023. Reporting quality assessment was conducted using the Consolidated Health Economic Evaluation Reporting Standards 2022 checklist. Of 5,180 screened articles, 17 were included in the final synthesis, with most from the United States and focused on tertiary prevention delivered to offenders. While the intervention components varied across studies, all demonstrated promising and cost-effective results. The findings highlight a small but growing body of economic evidence for child sexual abuse/exploitation interventions. The existing economic evaluation evidence is dominated by tertiary prevention, which focuses on offenders and child victims and highlights the need for more research and action on primary and secondary preventative interventions for general and at-risk populations.

## Introduction

Child sexual exploitation is a significant social problem, which is a form of child sexual abuse, and yet there is no consistent or universally accepted definition (Laird et al., 2022). Child sexual exploitation involves a child being “sexually exploited for money, power or status” (NSPCC, 2023). With a power imbalance in place, child sexual exploitation emphasizes an exchange whereby the child is enticed or forced into “sexual activity in return for something received by the child and/or those perpetrating or facilitating the abuse” (Beckett & Walker, 2016). For example, it could include a child receiving or believing that they will receive alcohol, drugs, accommodation, money, or gifts in exchange for sexual activities (Greijer & Doek, 2016). During this time of social media and technological innovations, online child sexual exploitation includes “the production of images of such abuse and the sharing of those images” (EUROPOL, 2022).

Since child sexual exploitation is a form of child sexual abuse, it is often necessary when reviewing existing evidence to look at the broader concept of child sexual abuse. According to the World Health Organization (WHO), child sexual abuse is defined as “the involvement of a child in sexual activity that he or she does not fully comprehend, is unable to give informed consent to, or for which the child is not developmentally prepared and cannot give consent, or that violates the laws or social taboos of society. Child sexual abuse is evidenced by this activity between a child and an adult or another child who by age or development is in a relationship of responsibility, trust or power, the activity being intended to gratify or satisfy the needs of the other person” (World Health Organization, 1999). However, the definition of child sexual abuse widely varies across the literature. Mathews and Collin-Vézina (2019) have developed a conceptual framework and defined child sexual abuse by including the following elements: an act that either provides sexual gratification to another person or is experienced by a child as a sexual act where a child is either unwilling or unable to consent and occurs while exploiting the vulnerability of the victim who is in a position of inequality.

Estimates of the global prevalence of child sexual abuse range from 8% to 31% and 3% to 17% among girls and boys, respectively, across different parts of the world (Barth et al., 2013). A meta-analysis published in 2015 has estimated global child sexual abuse at 7.8% and 18% in boys and girls (Stoltenborgh et al., 2015). In a nationally representative sample in Australia, 28.5% of respondents aged 16 years or older reported having experienced child sexual abuse (Mathews, 2023). In the United States in 2021, more than 29.3 million suspected child sexual exploitation were reported to the “National Centre for Missing and Exploited Children’s CyberTipline” a 35% increase from 2020 (National Center for Missing & Exploited Children [NCMEC], 2023). In Australia, the “Australian Centre to Counter Child Exploitation child protection triage unit” received more than 33,000 reports of online child sexual exploitation in 2021 (ACCCE, 2023). These figures show that child sexual abuse, inclusive of child sexual exploitation, is a widespread issue.

The WHO has identified child sexual abuse among 24 global risk factors, contributing to 0.6% of the global disease burden (World Health Organization, 2009). Child sexual abuse can result in both short and long-term adverse effects. Children with lived experience of child sexual abuse (i.e., any individual under the age of 18) can suffer from mental health conditions such as anxiety and depression, post-traumatic stress disorder, substance disorders, and behavioral issues (Olafson, 2011). Similar detrimental effects have been reported for child sexual exploitation, such as anxiety, depression, self-harm, and insecurity (Levine, 2022). Child sexual abuse can impact the quality of life throughout the life span, leading to reduced performance in education, employment, family life, and relationships (De Jong et al., 2015). Therefore, prevention is imperative. There are three levels of prevention: primary (pre-abuse), secondary (early detection and intervention for those at risk), and tertiary (post-abuse).

Research has shown the importance of incorporating a public health approach, which involves multidisciplinary actions at the community level to prevent child maltreatment, including sexual abuse (Herrenkohl et al., 2019). Family-based prevention interventions, such as home visiting, are effective in preventing other forms of child maltreatment (Holzer et al., 2006), but there is no evidence as to whether these are equally effective in preventing child sexual abuse. Similarly, while there is evidence that school-based interventions effectively improve knowledge, there is no evidence to support whether these lead to reduced child sexual abuse (Russell et al., 2020).

While child sexual abuse interventions are relevant to raise awareness and prevent child sexual exploitation, they do not fully address the unique dynamics of child sexual exploitation. Interventions targeting child sexual exploitation must consider several unique aspects that differentiate them from those addressing other types of child sexual abuse. For example, child sexual exploitation often involves the child receiving something in exchange for sexual activities, such as money, gifts, or even basic needs (NSPCC, 2023). Interventions must address these underlying needs to reduce the risk of such exchanges (UNICEF, 2020). Moreover, child sexual exploitation often occurs online, involving sophisticated grooming techniques, and therefore, interventions should include digital literacy and online safety education, as well as collaboration with tech companies to monitor and prevent online exploitation (Mannarino & Cohen, 2021).

To determine the best interventions, evidence on intervention costs and cost-effectiveness alongside outcomes is needed. This information would support decision-makers in using available resources and budgets to implement sustainable, effective interventions with optimum value for money. Recently, the Australian Research Council funded a project called “Disrupting Child Exploitation (DICE)” to develop a multiagency response to address child sexual exploitation.

There has been no systematic review synthesizing evidence from previous studies on the cost-effectiveness of prevention interventions for child sexual abuse and exploitation. Therefore, as part of the DICE project, this review aims to systematically synthesize the evidence for the cost-effectiveness of interventions for preventing child sexual abuse or exploitation. The key focus is to summarize the evidence on any prevention intervention (i.e., individual or community-based) for child sexual abuse and exploitation, identifying cost categories used for costing an intervention and any gaps in the literature to facilitate future research.

## Methods

### Search Strategy

The review team developed the search strategy, and six databases were searched via EBSCOhost: CINAHL Complete, Global Health, Medline Complete, SocIndex with Full Text, Social Work Abstracts, and APA PsycExtra. Campbell Collaboration and Cochrane Library (a collection of six databases, which includes access to the Cochrane Database of Systematic Reviews, Cochrane Central Register of Controlled Trials, Cochrane Methodology Register, Database of Abstracts of Reviews of Effects, Health Technology Assessment Database, and NHS Economic Evaluation Database) were also searched. The first 10 pages of a Google search of known websites (Supplemental Appendix 1) were conducted to identify gray literature. Various search terms for intervention, child sexual abuse and sexual exploitation, and economic evaluation were utilized (Supplemental Appendix 1). Although the initial target was to explore evidence on child sexual exploitation, upon a preliminary exploratory search, minimal evidence was found on child sexual exploitation. Therefore, our search included both child sexual exploitation and the broader concept of child sexual abuse.

### Selection Criteria

Studies were included if they were cost or economic evaluation studies of prevention interventions of child sexual abuse or sexual exploitation. Economic evaluation is defined as being a comparison of two (or more) options and presenting data on both inputs (costs) and outcomes, while a cost analysis presents a comparison of options but only in terms of cost (no outcome data) (Drummond et al., 2015). Of the various forms of economic evaluation, cost-benefit analysis compares the costs and benefits of an intervention in monetary terms. In contrast, cost-effectiveness analysis compares costs in monetary terms to outcomes expressed in natural units such as life years gained or symptom-free days, providing results as cost per unit change in outcome (Drummond et al., 2015). Cost-utility analysis is a special form of cost-effectiveness, where the outcome is a utility-based population health measure, such as quality-adjusted life years. In cost-consequences analysis, costs, and outcomes are separately presented but are not statistically compared (Torrance, 1997). Break-even analysis is a form of cost-benefit analysis that estimates the point at which the value of outcomes is equivalent to total costs (Hatch et al., 2017). Economic evaluation studies are conducted using data from a controlled trial or using economic modeling where data inputs for costs and effects are from different sources (Drummond et al., 2015).

Community or individual-based interventions targeted at children (even if the sample contained individuals over 18, as long as the sample contained children under 18), carers of children, or perpetrators/offenders, and studies from any country published until April 2023 were included. Non-English articles needed to have an abstract published in English to be included in the title and abstract screening to review eligibility. Studies were excluded if they did not meet the above criteria or focused on other areas of abuse (i.e., physical abuse, emotional abuse, neglect, and domestic violence) or noneconomic evaluations (e.g., effectiveness or efficacy).

### Study Selection and Data Extraction

All references were imported to Endnote 20 (The EndNote Team, 2020), and then imported to Covidence (Covidence, 2020). Duplicates were removed from Endnote and subsequently from Covidence. The first author (reviewer SWAD) completed the search, and then two reviewers independently screened the titles and abstracts of all articles selected by the search strategy (reviewers SWAD and MRA). Discrepancies were discussed between the two reviewers, and any disagreement was resolved with a third reviewer (reviewer LG). Three reviewers independently conducted the full-text screening of papers that met inclusion criteria on the initial screen of title and abstract (reviewers SWAD, GB, and JG). Discrepancies were discussed and resolved with an additional reviewer (reviewer LG). In the full-text screening, when an article met two or more exclusion criteria, it was excluded for the first reason met based on the following order: (a) no specific target on child sexual abuse/exploitation, (b) not an economic evaluation, (c) only an abstract, and (d) commentary/workshop summary/news article/presentation.

The information extracted from included studies was: prevention type (primary, secondary, or tertiary); target population; intervention; type of economic evaluation (cost-effectiveness analysis, cost-utility analysis, cost-benefit analysis, or cost analysis); comparator; study perspective; study time horizon; country; outcomes included and outcome measures; cost (components, costing approach, and costs results); and cost-effectiveness results. The first author extracted data from included studies, which the second author cross-checked.

### Quality Assessment

Two authors (reviewers SWAD and LG) independently assessed the reporting quality of included studies using the “Consolidated Health Economic Evaluation Reporting Standards” (CHEERS) 2022 checklist (Husereau et al., 2022). Any discrepancies were resolved after a discussion with another reviewer (reviewer MRA). CHEERS checklist consists of 28 questions on the title, abstract, introduction, method, results, discussion, and other relevant information such as funding and conflict of interest. Though CHEERS is not a scoring checklist, we chose to score the studies as done in previous literature that used the same tool to present a simple overall measure of reporting quality, allocating one point for “Yes” when an applicable criterion is met. The quality of the studies was rated as high, average, and poor based on the proportion of criteria met as >75%, 50% to 75%, and <50%, respectively (Althuwaibi et al., 2023).

### Data Analysis

To allow comparison of the cost data presented across studies, all costs were converted to 2023 U.S. dollars using the CCEMG-EPPI-Centre cost converter online tool (Cochrane Campbell Economic Methods Group and the Evidence for Policy and Practice Information and Coordinating Centre [CCEMG–EPPI], 2010). When the year of currency for cost data was not reported (*n* = 5), it was estimated using the year before the publication date. Although formal meta-analysis would be the preferred method to synthesize cost and cost-effectiveness results, our preliminary searches (to inform the review search strategy) suggested a highly divergent or heterogeneous evidence base with variation across population, intervention, comparator, outcome, and economic evaluation type. We, therefore, chose narrative synthesis to summarize the findings of the included studies. Based on the extracted data, the studies were grouped by similar themes and reported accordingly.

## Results

This systematic review is reported following PRISMA guidelines (Page et al., 2021) and registered in PROSPERO (CRD42023416494) (Wanni Arachchige Dona et al., 2023).

### Search Results and Study Characteristics

**Figure 1.**
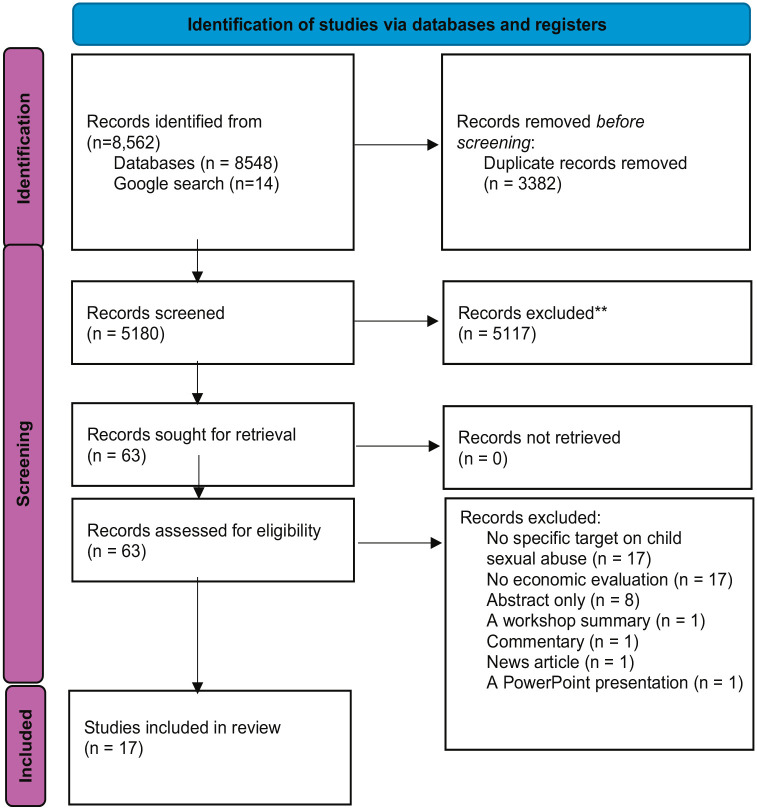
PRISMA flow diagram. **Did not meet inclusion criteria at the title and abstract screening or focused on other areas of abuse (i.e., physical abuse, emotional abuse, neglect, and domestic violence) or noneconomic evaluations (e.g., effectiveness or efficacy).

The search resulted in 8,562 records (8,548 from databases and 14 from Google search). After duplicates were removed, 5,180 records underwent title and abstract screening. Sixty-three non-English records were screened in this stage, but all were excluded as none met the inclusion criteria. Full-text screening was conducted for 63 articles, of which 17 were included in the final synthesis (Figure 1).

Of 17 studies, most (*n* = 14) were child sexual abuse interventions, with 3 child sexual exploitation studies. Fourteen studies focused on tertiary prevention, two primary prevention, and one covered both secondary and tertiary prevention levels (Table 1).

**Table 1.** Study Characteristics.

Author (Year)	Intervention and Comparator	Target Population (Intervention vs. Comparator)	Country or Region (Setting)	Economic Evaluation	Economic Perspective and Time Horizon	Cost Year or Currency Year	Discount	Outcomes	Cost Components/Cost Items	Results (US$ 2023)
Primary prevention										
Carrington et al. (2019)	Educational intervention vs. no intervention	Children and young people (*N* = 8,530)	Australia, Far North Queensland	Cost analysis (trial-based)	NR, 1 year	2016–2017	Not applicable	Not applicable	Personnel accounted for 74% of the total cost (a full-time Sergeant and a part-time Senior Constable) Operational expenses accounted for 26% of the total cost (travel, promotional materials, and other consumables)	Average cost per participant $22
Shipe et al. (2022)	School-based CSA prevention interventions	Elementary school children (*N* = 14,235)	United States	Cost analysis (trial-based)	NR, NR	2020 (assumed)	Not applicable	Not applicable	Program activities and materials (facilitator training, trainee expenses, video equipment, fidelity monitoring, activity booklets, extra materials) Personal and travel (facilitator, site coordinator, fringe, travel) Indirect costs (overheads, equipment, and materials) Facilities (physical space for facilitator training) Societal (required caregiver inputs)	Average direct cost per participant per year $23 (95% CI [68.06, 36.16]) Average caregiver time cost per student $26 per year
Mix of Secondary and tertiary prevention										
Shuker (2013)	2-Year “Safe Accommodation Project”	Children under age 18 at risk or victims (*N* = 663)	United Kingdom	A break-even analysis (trial-based)	NR, NR	2012	3.50%	Going missing, engagement with education, contact with the youth justice system, and placement stability	The running of specialist placements, including the costs of direct work, training (unrelated to specialist placement delivery), and evaluation costs.	Net benefit $362,578
Tertiary prevention										
Donato et al. (1999)	Offender treatment program	Offenders	Australia	CBA (trial-based)	NR, NR	1998	NR	Avoided cost of recidivism	Cos of “generic” in-prison program (average figure from a range of in-prison programs)	Potential economic savings could range from around $97,212 to over $1 million per 100 treated prisoners Average cost per victim of child sex abuse $22,228 A 10-percentage point reduction in recidivism rates, the net economic benefits range from $764,052 to $3.4 million
Donato and Shanahan (2001)	Rehabilitation	Offenders	United States (Massachusetts)	CBA (trial-based)	NR, NR	1990	NR	Net expected benefit per treated prisoner	NR	An expected net benefit per treated prisoner $64,316
Prentky and Burgess (1990)	Rehabilitation	Offenders (*N* = 129)	United States	CBA (model-based)	Societal, NR	1990	NR	The recidivism rate	Pretrial investigation, trial costs (not specified)	Total projected cost (original case plus a single re-offense) is $130,246 less for a treated than for an untreated child molester Total cost per offense $351,209
Shanahan and Donato (2001)	CBT programs	Offenders	Australia (South Australia)	CBA (model-based)	Societal, NR	1998	NR	Tangible benefits	Program cost for a “generic” program using an average figure derived from several programs The approximate average annual operating costs per treated offender	The economic benefits range from an expected net loss of $11,098 to an expected net benefit of $64,598 per treated prisoner depending on the monetary valuation placed upon intangible costs of child sex abuse and the efficacy of the treatment program
Jülich (2001)	Restorative justice pilot vs. Traditional current criminal justice system	Adult survivors of child sexual abuse, offenders, community	New Zealand	CBA (model-based)	NR, 20 years	2000	10%	Reduced cost to the criminal justice system, denunciation of crime, a decrease in the use of imprisonment and a decrease in the length of the prison sentence imposed, reduced offending	Start-up cost (e.g., protocol development, development of process, and development of educational material and information packs) and operating cost	Potential savings per victim $37,548 Annual net present value $92,995 Benefit–cost ratio 0.93 in year 1, 0.97 in year 2, and 0.98 in year 3, 0.99 in years 4 and 5, 1.00 in years 6 and 7
Borduin and Dopp (2015)	MST vs. usual community services	Juvenile sexual offenders (*N* = 24) vs. usual community services (*N* = 24)	United States (Missouri)	CBA (model-based)	Taxpayer’s perspective, 8.9 years following treatment	2013	3%	Benefit–cost ratio	Costs associated with criminal offenses pertaining to (a) taxpayer expenses, (b) tangible losses to victims, and (c) intangible losses to victims Personnel costs (e.g., therapist salaries, supervisor salaries, payroll taxes, employee health insurance, professional fees), non-personnel personnel expenditures (e.g., supplies, rent, utilities, maintenance, parking, depreciation), training and licensing costs, cell phone service contracts, and mileage reimbursement to therapists for travel related to providing services	14.41 Benefit–cost ratio for the taxpayer Every $1 (US$1.20) spent on MST for problem sexual behaviors recovered $58.51 in savings to taxpayers and crime victims $15,277 per youth for the intervention
Cotton (1991)	Community-based Juvenile sex offender treatment program	Juvenile sex offenders (*N* = 227)	United States (California)	Cost analysis (trial-based)	NR, NR	NR	Not applicable	Not applicable	Treatment services provided included; individual therapy sessions, group therapy sessions, case management meetings, network meetings, collateral sessions with offender’s family, family/couples therapy, home visits for case management, other treatment sessions, psychological testing while in treatment, and skills groups	Average cost of $13,163 for full 20 months of the pilot program vs. $289,587 for 22 months CYA (CYA’s treatment facility and parole Annual cost per offender for the three projects averaged $7,898 which included both direct and indirect costs
Giles et al. (2021)	Rapport-based instigative interviews	Suspects of child sexual abuse (*N* = 25)	United States	CBA (model-based)	NR, NR	Assumed 2020	NR	Total cost savings attributable to predominant use of adaptive cooperative interviewing		$239–974 million (annual unit costs) increasing to $3–$12 billion (lifetime costs) for all CSA
Gospodarevskaya and Segal (2012)	CBT vs. no treatment	Children who were sexually abused (*N* = 8,841)	Australia	CUA (model-based)	Australian mental health care system, 1 and 30 years	2010/2011	5%	Incremental costs, ICER, and QALYs	Mental health professionals Medications	ICER for treatment methods remained less than $6,038 per QALY gained Cost per child for non-directive counseling $1,747 TF-CBT only $1,724 TF-CBT+SSRI $1,867
Pazderka et al. (2022)	Multimodal treatment program (BBR)	Children who were sexually abused	Canada (Alberta)	CBA (a SROI analysis model-based)	Societal, 5 years	Assumed 2021 as the previous year of publication	0.05	SROI ratio	Staff salaries, contract therapists therapy supplies, insurance, travel costs, meals/snacks, utilities and telephone, repairs and maintenance, onsite security	Each dollar spent on treatment results in an average cost savings of $12.09 over 5 years (sensitivity analysis suggests a range of 9.58–13.34). Average annual cost for an individual receiving services at the BBR is estimated at $15,542
McCrone et al. (2005)	Individual vs. group psychotherapy	Children who were sexually abused (*N* = 71; 35 for individual therapy, and 36 for group therapy)	United Kingdom (Tavistock and Camberwell)	CEA/or cost-consequences analysis more strictly (trial-based)	Mental health services providers, 20 sessions (individual) vs. 18 sessions (group therapy), follow-up in 1 and 2 years	Assumed 2004 as the previous year of publication	NR	Psychiatric symptoms, symptoms of post-traumatic stress disorder, and global functioning	Salary, employer superannuation, national insurance contributions, overheads, and capital costs.	Total mean costs of individual therapy were found to be $2,596 greater than for group therapy
Block et al. (2013)	Multiple interviews vs. single interview	Children who were sexually abused	Not specified (United States)	CEA (model-based)	NR, NR	2012 (Assumed)	NR	Criminal convictions Cost for an additional conviction	The additional resources required to conduct multiple interviews Additional law enforcement resources required to prosecute and incarcerate offenders	Multiple interviews would cost an additional $121,974 per criminal conviction A reduction of six offenders implies benefits of $731,844
Pro Brono Economics (2011)	Multiple Barnado’s interventions (not specified) vs. no intervention	Children (young people who have been sexually exploited) (*N* = 539)	United Kingdom	CEA (model-based)	Taxpayer perspective, lifetime	2010	3.5%	Primary outcome: reduction in the risk of sexual exploitation Other outcomes: • Reduced missing episodes • Reduced alcohol and drug abuse • Improved engagement in education, training, or employment • Reduced accommodation and housing need	Costing the intervention was done in three approaches to get unit cost supporting young people	Potential savings of $5.5 or $11 for every $1 spent Total savings per case after intervention vs. before intervention $31,984 Total savings per case after intervention vs. synthetic control group $64,128 The average unit cost of providing an intervention is estimated to be in the region of $3,627–$6,348 per intervention, with a central estimate of $5,292) If the risks increase in the absence of intervention, as the econometric model suggests, the average savings rise to $63,476 per case
Crivelaro et al. (2022)	Health promotion program	Adolescents and youngsters who were sexually exploited *N* = 2,195 (cycle 1) *N* = 1,179 (cycle 2)	Brazil	Cost analysis (trial-based)	Service provider perspective, 1 year for each cycle	2019	Not applicable	Not applicable	Direct costs according to the conceptualization of disease cost-of illness studies, including expenses with human resources and social costs, administrative items (water, electricity, rent, internet, telephone, etc), consumables, transport and accommodation (fares, per diems), educational material and training, and third-party services (information technology, development, and maintenance of software and hardware)	Annual average cost per participant between $ 4,077 and $29,580

*Note.* NR = not reported; SROI = social return on investment; CEA = cost-effectiveness analysis; CUA = cost-utility analysis; CBA = cost-benefit analysis; CYA = California Youth Authority; QALY = Quality-Adjusted Life Year; BBR = Be Brave Ranch; MST = multisystemic therapy; CBT = cognitive behavioral therapy; TF = trauma-focused; SSRI = selective serotonin reuptake inhibitors; CSA = child sexual abuse.

Studies were most commonly from the United States (*n* = 6), Australia (*n* = 4), and the United Kingdom (*n* = 4), with one study each from Brazil, New Zealand, and Canada. Eight studies were published within the last decade (2013–2023), while nine were published between 1990 and 2012. Study populations included children (*n* = 9), adult offenders (*n* = 5), juvenile offenders (*n* = 2), and adult survivors (*n* = 1).

The most common form of economic evaluation (*n* = 8) was cost-benefit analyses, followed by cost-effectiveness analyses (*n* = 2), cost-consequences analysis (*n* = 1), cost-utility analysis (*n* = 1), and break-even analysis (*n* = 1), with four studies presenting a cost analysis. Of the studies included in this review, nine were model-based and eight were trial-based studies.

Study perspective and time horizon were not reported in nine different studies; where reported, studies adopted a societal (*n* = 3), state/taxpayer (*n* = 2), healthcare system (*n* = 1), or provider/care system (*n* = 2) perspective and the time horizon varied from 1 to 30 years. In terms of the quality of reporting, 15 out of 17 studies were rated average or high, having reported 50% or more of the items on the CHEERS checklist (Supplemental Appendix 2). Two studies reported less than 50% of the items, with one of these studies published before 2000, that is, before the widespread use of reporting guidelines. All studies reported on 6 of the 28 items: abstract, background and objectives, setting and location, selection of outcomes where applicable, summary of main results, and the discussion item (study findings, limitations, generalisability, and current knowledge). No study met reporting criteria concerning health economic analysis plans or methods of subgroup analysis, and only about half of the studies reported methodology on discount rates, currency, price rate and conversions, and uncertainty.

### Primary Prevention

Two studies conducted cost analysis on educational programs to prevent child sexual abuse: a school-based and a community-based intervention (Carrington et al., 2019; Shipe et al., 2022). While the two interventions varied in the targeted population, setting, time duration, and intervention components (Table 1), both estimated the direct cost of the intervention per participant was about $22 to $23 per year. Both studies considered direct costs such as personnel, travel, and program materials in their cost analysis, with personnel (staff) cost reported as the most significant contributor to the total cost (74% in the study by Carrington et al., 2019). Shipe et al. (2022) reported an additional direct cost of carer time of $26 per year. The cost data were sourced directly from the trials, and the authors claimed that these interventions were promising and cost-efficient based on the available evidence on the total lifetime cost of victims.

### Secondary Prevention

No studies were found on interventions solely targeting secondary prevention. Only one study from the United Kingdom considered the cost-effectiveness of specialist placements for at-risk or victimized children with sexual exploitation or trafficking. These placements were incorporated with a prior 2-day professional training for staff (i.e., a mix of secondary and tertiary prevention) (Shuker, 2013). Costs attributable to running a specialist placement were considered, but the authors identified that some costs (e.g., management and administrative overheads) were not directly included in the analysis. Although the intervention indicated potential cost savings, the authors reported that there were limitations in identifying a suitable comparator and quantifying outcomes to assess effectiveness (and therefore whether the intervention was cost-effective) due to the small number of placements and their short durations, and the difficulties generalizing findings to all children affected by child sexual exploitation. Therefore, a break-even/cost analysis was conducted, where measurable outcomes were valued to identify potential savings. This found that placements could be cost-effective, assuming the outcomes would not change without the intervention (i.e., constant risk profile) and that the intervention was more likely to be cost-effective if it effectively targeted the intended population (i.e., children) and if children were more willing to engage with the intervention. The intervention cost per child or per professional training was not provided and could not be calculated as the cost was reported at an aggregated level. While the avoided costs were obtained from previous reports, there was no information on the source of the intervention cost. This 2-year project resulted in savings of $362,578 for the avoided “going missing” episodes from placements, engagement with education, and placement stability.

### Tertiary Prevention

Of tertiary interventions, six studies were for children who have been sexually abused, and one study was for adult survivors of child sexual abuse or exploitation. Seven studies were for offenders or suspects of child sexual abuse or exploitation.

### Offenders

Seven studies on offender-targeted interventions reported that they can be cost-effective. Of them, four studies were rehabilitation or behavioral therapy for sexual offenders in prison. Prentky and Burgess (1990) conducted a cost comparison, reporting a total projected cost (for an original case plus a single re-offense) as $130,246 less for treatment in a rehabilitation program than for an untreated offender, with 25% less recidivism (re-offending) for treated compared with non-treated offenders. As other studies reported (Donato & Shanahan, 2001; Donato et al., 1999; Shanahan & Donato, 2001), this study has limitations such as not including intangible costs (e.g., pain and suffering), which could be up to 10 times the magnitude of tangible costs, resulting in significant underestimation of victim costs (and the cost savings or benefits of reduced crime) if they were excluded (Donato & Shanahan, 2001). The academic debate prompted by this study highlights the importance of carefully considering the source of projected cost savings: whether from a reduction in high-cost activities such as shorter prison time or from improved outcomes from reduced crime (Donato & Shanahan, 2001; Donato et al., 1999). Donato et al. (1999) investigated cognitive treatment programs for those who have committed child sexual offenses and considered costs for victims, such as care and protection, health and education services, and law enforcement and justice services (e.g., police, legal, court, attorney general, and correctional services). They found that the intervention can be cost-effective if the recidivism rate is reduced by at least 6%, and if recidivism is reduced by 10%, the (aggregate) net economic benefits range from $764,052 to $3.4 million. Another study, comparing the traditional justice system with the restorative justice program targeted at victims, offenders, and the community, reported that they could yield significant savings, with potential savings per victim of $3,754 through conferencing with them (Jülich, 2001).

Two studies included community-based treatment interventions for juvenile sex offenders (Borduin & Dopp, 2015; Cotton, 1991). The average cost per offender varied between $13,163 (Cotton, 1991) and $15,277 (Borduin & Dopp, 2015) for family or community-based interventions. Cost-benefit analysis of an intensive family and community-based multisystemic therapy (MST) for problematic sexual behaviors was found to be highly cost-effective, with $58 in savings to taxpayers and crime victims for every $1 spent (Borduin & Dopp, 2015). The costs were reported at an aggregated level, and the average cost of MST was $15,277 per youth. The intervention has resulted in long-term economic benefits through avoiding future crimes (Borduin & Dopp, 2015). Similarly, a cost analysis study assumed that another community-based juvenile sex offender treatment program with an annual cost of $7,898 per offender, which was only 6% of the cost of traditional institutionalized treatments, such as incarceration, specialized treatments, parole, and youth centers, could be cost-effective compared with the traditional jurisdictional method, due to vastly reduced costs and assumed equivalent outcomes (Cotton, 1991).

Giles et al. (2021) found potential economic benefits of rapport-based interviews with suspects of child sexual abuse, reporting potential annual cost savings of $239 to $974 million, increasing to lifetime cost savings of $3 to $12 billion from reduced recidivism of all child sexual abuse. However, this study claimed that there might be more economic benefits and a full cost-benefit analysis is needed.

### Children

While the interventions for children who were sexually abused or exploited varied across studies, all of them were found to be promising and favorably cost-effective. Three studies focused on various treatment programs (e.g., psychotherapy treatments and multimodal treatments) that were found to be cost-effective (Gospodarevskaya & Segal, 2012; Pazderka et al., 2022). Group treatments were more cost-effective than individual treatments for children to overcome the consequences of child sexual abuse/exploitation (McCrone et al., 2005). The cost categories were consistent, varying only slightly across the three studies. A social return on investment of a multimodal treatment program in Canada considered direct costs, such as therapists, other staff, therapy supplies, administrative costs, meals, accommodation, and onsite security, as the intervention was like a camp setting (Pazderka et al., 2022). The annual cost per person for holistic treatments in a more camp-like setting was $15,542 (Pazderka et al., 2022). A challenge in estimating the cost per child was that children starting the treatment program at different times of the year, location distance, and the nature of the continuation of the treatment (Pazderka et al., 2022). A Markov-model-based cost-utility analysis comparing different psychotherapy treatments to no treatment included direct costs of resource use, such as mental health professionals and medications, obtained from administrative databases (the Australian MBS and PBS) (Gospodarevskaya & Segal, 2012), assuming MBS cost for psychiatrists and psychologists would cover patient time and overheads. They reported that each of the three treatments (non-directive counseling, cognitive behavioral therapy [CBT], and CBT plus drug treatment) would be a better investment than no treatment from the Australian mental health system perspective, at an estimated annual cost per child of $1,747, $1,724, and $1,867, respectively (Gospodarevskaya & Segal, 2012). McCrone et al. (2005), who compared individual therapy to group therapy, found that the mean cost of individual therapy was $2,595 greater than that of group therapy which has similar outcomes from both pathways. However, they claimed that the costs linked to waiting time for children to enter a group therapy were not included, and potential delays should be considered when trading off between potential savings and urgency of treatment for those in need.

Interviewing children multiple times has increased the disclosure and the possibility of an offender being convicted by 6.1%. Interview-based interventions are considered to be cost-effective, but interviews can be equally costly if not implemented correctly (Block et al., 2013). Block et al. (2013) conducted a cost-effectiveness analysis on multiple forensic interviews and reported that multiple interviews would cost an additional $121,974 per criminal conviction. This cost was only for the interview itself, and other costs that have not been considered might include costs for law enforcement and social services. A reduction of six offenders was reported to imply benefits of $731,844. However, the authors have not considered the cost of false convictions, and costs in terms of reliving trauma and negative impact to mental health, which could substantially cost society (Block et al., 2013).

Two studies were on health promotion programs for children who were sexually exploited. A cost analysis of a health promotion program reported an annual average cost per participant between $4,077 and $29,580 depending on the mix of interventions delivered (Crivelaro et al., 2022). This intervention had multiple areas, focusing on health (i.e., diagnosis), family involvement, technical education and mainstream education, and employability. All the costs were direct costs at an aggregated level, including expenses with human resources, administration, consumables, transport, program materials, and other third-party services (e.g., IT). Pro Brono Economics (2011) performed a cost-effectiveness analysis on the multiple interventions implemented by Barnardo’s (a not-for-profit organization in the United Kingdom) and reported that the combination of those interventions brings benefits to the taxpayer which outweighs the costs substantially, with a potential savings of $5.50 or $11 for every $1 spent. The interventions were delivered to children who had experienced sexual exploitation, which included allowing access to services, allocating a key worker, and advocacy for children, and outcomes were measured in terms of “going missing” episodes, engagement in education, substance abuse, and accommodation needs, risk of sexual exploitation, and relationship with parents/friends. The average saving per case for the intervention was $63,476 if the risks increased in the absence of intervention. Barnardo’s has implemented multiple interventions, and their cost were not necessarily distinguishable; therefore, they separated the cost of the sexual exploitation-related services from other work undertaken at Barnado’s based on the total expenditure at Barnado’s and funding from other sources for sexual exploitation interventions (Pro Brono Economics, 2011).

## Discussion

To the best of our knowledge, this is the first systematic review aimed to investigate the cost-effectiveness evidence of child sexual abuse and exploitation prevention interventions. Only half the studies were full economic evaluations, all of which were model-based, which is sensible as the long-term effects of child sexual abuse go beyond those that can be measured in the duration of a trial. It was evident that all types of prevention interventions (i.e., primary, secondary, and tertiary) were found to be promising and possibly cost-effective (requires increased funding but “good value for money”) or even cost-saving (saves more than it costs). The literature on economic evaluation of preventing child sexual abuse has been growing, but there is still scarce evidence on costs and cost-effectiveness for interventions addressing child sexual abuse and exploitation.

Out of the three studies on interventions that specifically targeted child sexual exploitation, one analyzed only the program costs (cost analysis) and two presented a full economic evaluation (i.e., analyzed costs compared to outcomes). The cost analysis was of a health promotion program which included assessing healthcare needs and interventions to improve the bond between families and communities, education, and employability for sexually exploited young people (Crivelaro et al., 2022). This program targeted young people aged 16 to 24 across 18 Brazilian states. Seventy percent of these young people were female but all were of similar religions. The two economic evaluation studies found that interventions aimed at addressing child sexual exploitation could be cost-effective and bring savings that outweigh the costs (Pro Brono Economics, 2011; Shuker, 2013). One study analyzed a specialist placement initiative called the “Safe Accommodation Project” for at-risk or victimized children in the United Kingdom (Shuker, 2013). The young people referred to this program were those between the ages 13 and 17, and most were female (90%). The other study analyzed a combination of interventions, including initiatives to increase access to services, allocate a key worker, and advocate for young people who had experienced sexual exploitation (Pro Brono Economics, 2011). The model inputs for the analysis of the population were that the age of those receiving the interventions was younger than 18, with 85% female and 75% identifying as White British. Thus, the findings highlight limited cost-effectiveness evidence on community interventions targeting the general population for primary prevention or targeting at-risk populations for secondary prevention.

Theoretically, the first use of economic evidence is to estimate the cost of implementing interventions. Comparing costs across interventions is challenging due to the differences in prevention approaches and program components. The variety of studies in this review demonstrates that cost estimates vary according to the cost components included in a study, with the most common components being the staff time, space, and materials required to plan and deliver an intervention, with staff costs often forming the major cost component. There was consistency across studies on the inclusion of these common “direct costs,” such as health professionals and other staff, therapy supplies, administrative costs, meals, and accommodation. However, findings in this review highlight additional cost components for consideration, such as including child and carer time costs (Shipe et al., 2022). In addition, many studies did not account for intangible costs, such as costs incurred due to pain and suffering, leading to underestimation. Other studies demonstrate the impact of intervention setting for per-participant costs, for example, the increased costs that arise from increased travel costs and smaller population size in a rural area (Shipe et al., 2022).

The outcomes side of economic evaluation evidence weighs up intervention costs against the value of outcomes achieved. Studies in this review demonstrate an overall limitation due to the lack of evidence of intervention effectiveness, with some studies basing cost-effectiveness estimates on low-quality evidence of intervention outcomes (Donato et al., 1999; Shanahan & Donato, 2001; Shuker, 2013). Studies by Prentky and Donato emphasized the importance of specifying, which outcomes are included, notably whether intangible costs such as the pain and suffering associated with abuse are included in estimates of the cost savings of an intervention (Donato & Shanahan, 2001; Prentky and Burgess, 1990).

This review highlights important economic evaluation evidence to support implementing child sexual abuse or sexual exploitation prevention interventions at the tertiary level, targeting offenders and children who were affected. Most interventions were based on tertiary prevention delivered to offenders. This is consistent with the literature, which states that traditionally, the focus had been to address child sexual abuse through victim-targeted clinical interventions and offender-targeted interventions, but that a primary/secondary prevention focus is now required as it is more critical to prevent abuse before it happens through public health approaches and changes in social and educational policies (Letourneau et al., 2014). While primary interventions show promising results, there is a need for far more research on both the effectiveness and cost-effectiveness of primary prevention at the community level to determine the value for money credentials of primary prevention intervention at the community level for child sexual abuse and exploitation.

In terms of reporting quality of studies, most studies in this review had a satisfactory reporting quality. While reporting quality was inconsistent across the years, most unsatisfactory level reporting was found from studies published before 2000. As strengths, this review searched and included both peer-reviewed publications and gray literature, and studies included without any limitations on the country and published year. However, there are several limitations, including our failure to incorporate non-English studies with no English abstract and the inability to conduct meta-analysis due to the heterogeneity across studies in terms of populations, interventions, comparators, outcomes, and economic evaluation design. The evidence to date is largely representative of at-risk populations in high-income countries, and thus, the findings cannot be generalized to other contexts due to the lack of diversity in ethnicity, culture, and socioeconomics. Most of the study samples included child victim-survivors and adult offenders, while only a few studies covered adult survivors of child sexual exploitation and juvenile offenders. There is a critical need for future studies to include diverse population samples and consistent reporting of sample characteristics (e.g., sociodemographic).

Moreover, the findings advocate for the implementation and expansion of interventions for child sexual abuse or exploitation, emphasizing the importance of detailed and more robust evidence of the effectiveness. Future research should aim to address existing gaps: (a) the lack of studies on primary and secondary prevention, (b) the underreporting of key cost components such as intangible costs (e.g., costs associated with pain and suffering), (c) inconsistencies in reporting, and (d) the lack of additional cost components (e.g., child or carer time) apart from staff costs.

## Conclusion

This review found that prevention interventions to reduce child sexual abuse and child sexual exploitation at any prevention level have been assessed as promising and likely to have good value for money. To enhance our understanding of the value and effectiveness of these interventions, it is imperative to conduct rigorous studies, adopt various intervention approaches, and include diverse population samples. Most studies have considered the direct costs of the intervention in the evaluation, and it is important to consider costs and benefits from both tangible and intangible aspects to realize the cost-effectiveness of an intervention. By doing so, we can better inform policy decisions and allocate resources to protect children and support survivors more effectively.

**Table:** Critical Findings.

1.	Any level of prevention intervention for child sexual exploitation has been assessed as promising and likely to be cost-effective.
2.	A small but growing body of economic evidence for child sexual abuse/exploitation interventions is evident.

**Table:** Implications for Practice, Policy, and Research.

1.	Detailed and more robust cost-effectiveness analysis should be incorporated when implementing interventions for child sexual abuse or exploitation prevention.
2.	Future studies should aim to address existing gaps of lack of studies on primary and secondary prevention of child sexual abuse or exploitation.
3.	Future studies should aim to address the gap of underreporting and inconsistencies in key cost components of cost-effectiveness studies.

## Supplemental Material

sj-pdf-1-tva-10.1177_15248380241284782 – Supplemental material for Economic Evaluation of Prevention Interventions for Child Sexual Exploitation or Child Sexual Abuse: A Systematic Review Supplemental material, sj-pdf-1-tva-10.1177_15248380241284782 for Economic Evaluation of Prevention Interventions for Child Sexual Exploitation or Child Sexual Abuse: A Systematic Review by Sithara Wanni Arachchige Dona, Genevieve Bloxsom, Julie Green, Mary Rose Angeles, Cathy Humphreys and Lisa Gold in Trauma, Violence, & Abuse
