# Supplementary material for: Economic Evaluation of Prevention Interventions for Child Sexual Exploitation or Child Sexual Abuse: A Systematic Review
Source: Trauma Violence Abuse. 2024 Oct 10;26(3):546–59. doi: 10.1177/15248380241284782 (PMC12145467; doi:10.1177/15248380241284782)
Supplement: sj-pdf-1-tva-10.1177_15248380241284782 – Supplemental material for Economic Evaluation of Prevention Interventions for Child Sexual Exploitation or Child Sexual Abuse: A Systematic Review [file sj-pdf-1-tva-10.1177_15248380241284782.pdf]

Appendices

Economic evaluation of prevention interventions for child sexual exploitation or child sexual abuse: a systematic review

Sithara Wanni Arachchige Dona, Genevieve Bloxsom, Julie Green, Mary Rose Angeles, Cathy Humphreys, Lisa Gold

Appendix 1: Search Strategy

	**Search terms/concepts**
**Databases**	
Concept 1: Interventions (title OR abstract search)	Intervent* OR program* OR train* OR promot* OR support* OR initiative* OR standard* OR approach* OR educat*OR technique* OR protocol* OR strateg* OR prevent* OR OR service* OR polic* OR treatment
Concept 2: Child (title OR abstract search)	Infan* OR child* OR teen* OR youth* OR adolesc* OR minor* OR toddler* OR baby OR babies OR young OR "young person*" OR "young people" OR juvenile* OR girl* OR boy* OR "school age*" OR kid* OR underage OR under-age<br>OR (MH "Child+") OR (MH "Adolescent")
Concept 3: sexual exploitation/abuse (title OR abstract search)	((sex*) N3 (exploit* OR abus* OR harass* OR trade* OR viol* OR assult* OR victi* OR slavery OR maltreat* OR coerc* OR manipulate* or offend*)) OR CSE OR CSA OR trafficking OR groom* OR prostit* OR camming OR sext* OR porn* OR molestation OR paedophile OR pimping OR "child exploitation material*"<br><br>OR (MM "Child Abuse, Sexual") OR (MM "Child Advocacy")
Concept 4: economic evaluation (title OR abstract search)	cost* OR invest* OR econom* OR financ* OR value OR expenditure* OR spending* OR expense* OR resource* OR "net benefit*" OR "cost-utility" OR "cost-benefit" OR "cost-effectiveness" OR "vale-for-money" OR CBA OR CEA OR CUA<br>OR (MH "Cost-Benefit Analysis") OR (MH "Cost Control+") OR (MH "Cost Savings") OR (MH "Cost-Effectiveness Analysis") OR (MH "Costs and Cost Analysis+") OR (MH "Economics+")
	Concept 1 AND concept 2 AND concept 3 AND concept 4
**Google search**	

General search	("economic evaluation" OR "cost effectiveness" OR "cost benefit" OR “cost utility” OR “net benefit” OR “cost analysis”) ("child sexual abuse" OR "child sexual exploitation" OR “child sexual”)
Google search in Site:.gov. and Site:.gov.au 10 pages	("economic evaluation" OR "cost effectiveness" OR "cost benefit" OR “cost utility” OR “net benefit” OR “cost analysis”) ("child sexual abuse" OR "child sexual exploitation" OR “child sexual”) (site:.org.au)
Google search in Site:.gov.ca 10 pages	("economic evaluation" OR "cost effectiveness" OR "cost benefit" OR “cost utility” OR “net benefit” OR “cost analysis”) ("child sexual abuse" OR "child sexual exploitation" OR “child sexual”) (site:.gov.ca)
Similar search, using<br>Site:.org.uk<br>Site:.org.ca<br>Site:.org.nz<br>Site:.org.ie<br>to capture all relevant websites such as:<br>https://www.barnardos.org.uk<br>https://aifs.gov.au<br>https://www.dss.gov.au<br>https://www.anrows.org.au<br>https://www.unicef.org<br>https://www.aic.gov.au<br>https://www.scie.org.uk<br>https://www.frontiersin.org<br>https://www.un.org	("economic evaluation" OR "cost effectiveness" OR "cost benefit" OR “cost utility” OR “net benefit” OR “cost analysis”) ("child sexual abuse" OR "child sexual exploitation" OR “child sexual”) (site:. gov.ie)<br><br>("economic evaluation" OR "cost effectiveness" OR "cost benefit" OR “cost utility” OR “net benefit” OR “cost analysis”) ("child sexual abuse" OR "child sexual exploitation" OR “child sexual”) (site:. gov.uk)<br><br>("economic evaluation" OR "cost effectiveness" OR "cost benefit" OR “cost utility” OR “net benefit” OR “cost analysis”) ("child sexual abuse" OR "child sexual exploitation" OR “child sexual”) (site:. gov.ca)<br><br>("economic evaluation" OR "cost effectiveness" OR "cost benefit" OR “cost utility” OR “net benefit” OR “cost analysis”) ("child sexual abuse" OR "child sexual exploitation" OR “child sexual”) (site:. gov.nz)

Appendix 2: Reporting quality (the CHEERS 2022 checklist)

**Section/topic**	**Item No**	**Prentky et al., 1990**	**Cotton et al., 1991**	**Donato et al., 1999**	**Donato 2001**	**Julich et al., 2001**	**Shanahan et al., 2001**	**McCrone et al., 2005**	**Pro Bono Economics 2011**	**Gospodarevskaya et al., 2012**	**Block et al., 2013**	**Shuker 2013**	**Borduin and Dopp 2015**	**Carrington et al., 2019**	**Giles et al., 2021**	**Crivelaro et al., 2022**	**Shipe et al., 2022**	**Pazderka et al., 2022**
Title	1	1	0	1	1	1	1	1	1	1	1	1	1	1	1	1	1	1
Abstract	2	1	1	1	1	1	1	1	1	1	1	1	1	1	1	1	1	1
Background and objectives	3	1	1	1	1	1	1	1	1	1	1	1	1	1	1	1	1	1
**Methods**																		
Health economic analysis plan	4	0	0	0	0	0	0	0	0	0	0	0	0	0	0	0	0	0
Study population	5	1	1	0	0	0	1	1	1	1	1	1	1	1	1	1	1	1
Setting and location	6	1	1	1	1	1	1	1	1	1	1	1	1	1	1	1	1	1
Comparators	7	1	1	1	1	1	1	1	0	1	0	1	1	0	0	1	0	1
Perspective	8	0	0	0	0	0	0	1	1	1	0	0	1	0	0	1	1	1
Time horizon	9	0	0	0	0	1	0	0	1	1	0	0	1	1	0	1	0	1
Discount rate	10	0	0	0	0	1	0	0	1	1	0	1	1	0	0	0	0	1
Selection of outcomes	11	1	NA	1	1	1	1	1	1	1	1	1	1	NA	1	NA	NA	1
Measurement of outcomes	12	1	NA	0	0	0	1	1	1	1	1	1	1	NA	1	NA	NA	1
Valuation of outcomes	13	1	NA	1	1	0	0	0	1	1	0	1	1	NA	1	NA	NA	1
Measurement and valuation of resources and costs	14	1	0	0	1	1	1	1	1	1	1	0	1	1	1	1	1	1

Currency, price date, and conversion	15	1	0	1	1	1	1	0	1	1	0	1	1	1	0	1	0	0
Rationale and description of model	16	1	NA	NA	NA	1	1	NA	1	1	0	NA	1	NA	1	NA	NA	1
Analytics and assumptions	17	1	NA	1	1	1	1	1	1	1	1	1	1	NA	1	NA	NA	1
Characterising heterogeneity	18	0	NA	0	0	0	0	0	0	0	0	0	1	NA	0	NA	NA	0
Characterising distributional effects	19	0	NA	0	0	0	0	0	0	0	0	0	0	NA	0	NA	NA	0
Characterising uncertainty	20	0	0	1	1	0	1	1	1	1	0	1	1	0	0	0	1	1
Approach to engagement with patients and others affected by the study	21	0	0	0	0	0	0	0	0	0	0	0	0	0	0	0	0	0
**Results**																		
Study parameters	22	0	0	1	1	1	1	1	1	1	1	1	1	1	1	1	1	1
Summary of main results	23	1	1	1	1	1	1	1	1	1	1	1	1	1	1	1	1	1
Effect of uncertainty	24	0	0	1	1	0	1	0	1	1	0	1	1	0	0	0	1	1
Effect of engagement with patients and others affected by the study	25	0	0	0	0	0	0	0	0	0	0	0	0	0	0	0	0	0
**Discussion**																		
Study findings, limitations, generalisability, and current knowledge	26	1	1	1	1	1	1	1	1	1	1	1	1	1	1	1	1	1
**Other relevant information**																		
Source of funding	27	0	0	0	0	0	0	1	1	1	1	1	0	1	1	1	1	1

Conflicts of interest	28	0	0	0	0	0	0	0	0	1	0	0	0	0	0	1	1	1
Total number of “Yes”		14	7	14	15	15	17	16	21	23	13	18	22	12	15	15	14	22
Total score %		50%	33%	52%	55%	53%	61%	57%	75%	82%	46%	66%	79%	57%	53%	71%	67%	79%
Quality		Average	Poor	Average	Average	Average	Average	Average	High	High	Poor	Average	High	Average	Average	Average	Average	High

Note: 1=Yes, 0=No, NA=Not Applicable, Total score %= (Total number of “Yes”/ Total applicable items)%
